# Singlemoded THz guidance in bendable TOPAS suspended-core fiber directly drawn from a 3D printer

**DOI:** 10.1038/s41598-020-68079-y

**Published:** 2020-07-06

**Authors:** Wanvisa Talataisong, Jon Gorecki, Rand Ismaeel, Martynas Beresna, Daniel Schwendemann, Vasilis Apostolopoulos, Gilberto Brambilla

**Affiliations:** 1 Optoelectronics Research Centre, University of Southampton, Southampton, SO17 1BJ UK; 2 School of Physics and Astronomy, University of Southampton, Southampton, SO17 1BJ UK; 3 National Oceanography Centre, Southampton, SO14 3ZH UK; 4 Institute for Material Science and Plastics Processing, University of Applied Sciences Eastern Switzerland, 8640 Rapperswil, Switzerland

**Keywords:** Fibre optics and optical communications, Polymers

## Abstract

Terahertz (THz) technology has witnessed a significant growth in a wide range of applications, including spectroscopy, bio-medical sensing, astronomical and space detection, THz tomography, and non-invasive imaging. Current THz microstructured fibers show a complex fabrication process and their flexibility is severely restricted by the relatively large cross-sections, which turn them into rigid rods. In this paper, we demonstrate a simple and novel method to fabricate low-cost THz microstructured fibers. A cyclic olefin copolymer (TOPAS) suspended-core fiber guiding in the THz is extruded from a structured 3D printer nozzle and directly drawn in a single step process. Spectrograms of broadband THz pulses propagated through different lengths of fiber clearly indicate guidance in the fiber core. Cladding mode stripping allow for the identification of the single mode in the spectrograms and the determination of the average propagation loss (~ 0.11 dB/mm) in the 0.5–1 THz frequency range. This work points towards single step manufacturing of microstructured fibers using a wide variety of materials and geometries using a 3D printer platform.

## Introduction

Terahertz (THz) waves or T-waves occupy a window of electromagnetic waves with frequency ranging from ν ~ 0.1 THz to ν ~ 10 THz (corresponding to the wavelength range from λ ~ 30 µm to λ ~ 3 mm). Over the last decade, THz waves have been exploited in many applications owing to their unique characteristic such as ability to penetrate in most of dielectric materials and provide improved resolution when compared to micro- or millimeter waves. Security scanning, non-destructive testing and imaging are some of the most noteworthy application of THz technologies^1,2^. Because of their non-ionizing nature, THz waves can be used to detect organic tissue without causing damage, and can be safely applied for medical and biomedical sensing^3,4^. THz waves have great potential to be exploited for detecting chemicals, pharmaceuticals and biological agents, as the main rotational modes of many macromolecules have a strong absorption in the THz region^5,6^. They can also be used in wireless communications to increase data transmission, due to the large bandwidth of the THz band^7^, and in astronomy, to locate cold matter in space or for imaging applications in deep space^8,9^.

Although THz waves have shown strong potential for imaging and sensing, most of the THz systems are based on free-space optics that are quite delicate. Hence, a significant amount of research has focused on achieving low-loss and low-dispersion THz waveguiding. Metal wires were the very first material used in THz waveguides due to their low material absorption^10,11^: square and circular metallic waveguides have been demonstrated at the beginning of this century for very dispersive and low loss THz propagation^12^. In 2001, two thin metallic strips were used to construct a parallel plate THz waveguides for low loss and low group velocity dispersion^13^. The combination of metallic and polymer for fabricating THz waveguide was proposed in 2007 by coating silver and polystyrene inside a hollow-glass tube^14,15^. Low-loss guidance for TE mode at 2.5 THz was obtained using this configuration. These waveguides, however, suffer from a large field extension into air as well as from large bending loss. These drawbacks compromise their applications requiring low cross-talk between closely spaced lines (e.g. imaging) and remote operation, which require alternative solutions.

Polymer optical fibers were proposed for THz waveguiding because of their low cost, widely accessible materials, low-temperature processing, and relatively low loss (when compared to other dielectrics) in the THz spectral range^16,17^. However, most of polymers have high material absorption, leading to high loss in bulk polymer waveguides. To overcome this issue, hollow-core waveguides or fibers have been proposed: since the mode is mostly confined in the hollow air-core, the effect of material loss is minimal. The hollow-core can be easily filled with dry air fiber eliminating water vapors and their absorption^17^. Many types of hollow-core fibers were developed with different guiding mechanisms for THz guidance^18,19^, including photonic bandgap (PBG) and antiresonance (AR).

In 2008, the first hollow-core polymer fiber for THz guiding was made by drilling PMMA with a Bragg fiber structure^20^, provide a guiding structure with an overall loss (0.5 cm^−1^) 30–60 times smaller than the PMMA material loss at ν ~ 1.0–1.3 THz. The waveguide design was not optimized, and the total diameter was approximately 6 mm. Other THz Bragg fibers, reported in 2011^21,22^, were fabricated by depositing a layer of TiO_2_-doped polymer on a polymer film and then rolling it to create periodic layers around the hollow fiber core. These fibers had a total diameter of ~ 12 mm with estimated losses of ~ 0.1 cm^−1^ within the THz transmission window. Another category of hollow-core THz fibers is based on antiresonance^23,24^: light can be confined to the central air-core because of the antiresonant reflection of the guided wave at the membranes surrounding the core which behaves effectively as a Fabry–Perot cavity. A simple stacking technique was used to fabricate negative curvature antiresonant fibers^25,26^. Yet, the low interaction between THz waves and material necessary for low-loss propagation required a large core size (5–10 mm), thus a total fiber diameter (1–5 cm), which limited the fiber flexibility.

Index-guiding THz polymer waveguides have been reported using polymer ribbon structures^27^. A 2 cm wide and 150 µm thick high-density polyethylene (HDPE) was used for the first low-loss polymer ribbon waveguide. Solid-core microstructured waveguides based on the common hexagonal hole array exhibited losses of the order of 0.5 cm^−1^ when propagating over 2 cm^28^. In 2004, a 0.1 cm^−1^ loss over propagation lengths of 10 cm was achieved using polytetrafluoroethylene (PTFE)^29^. Although HDPE and PTFE have a low material attenuation (0.3 cm^−1^) compared with conventional polymers used for optical fibers, low loss operation is located only at ~ 1 THz. For this reason, extensive research has been carried out to identify polymers with lower loss over a wide range of frequencies in the THz regime: Zeonex and cyclic olefin copolymer (COC or TOPAS) have been so far the materials of choice to fabricate THz waveguide. TOPAS waveguides with the common hexagonal hole array along a propagating lengths of 9 cm were demonstrated in 2009^30^ and exhibited a loss smaller than 0.5 cm^−1^ over the frequency range of 0.1–3 THz. In 2011, a suspended-core THz fiber fabricated from a low density polyethylene (PE) was preliminary reported for the low loss guiding (0.02 cm^−1^) in the THz frequency range of 0.25–0.51 THz^31^. In the same year, the suspended-core THz fiber was fabricated using Zeonex^32^ showing losses of the order of 0.1 cm^−1^ over a fiber length of 7 cm, essentially matching the material loss.

In this paper, we report the design and fabrication of a suspended-core microstructured polymer optical fiber (SC-MPOF) for THz guiding. The extrusion technique and 3D printing have been combined to propose a novel low-cost fabrication method of long stretches of microstructured polymer optical fibers (MPOFs) with attractive transmission properties in the THz region. Compared with general extrusion, the dies used in this work were reduced three times in size to fit with any available 3D printer. By integrating fiber drawing and 3D printing, the methodology proposed in this manuscript represents a simple and low-cost alternative to the use of fiber drawing tower. The combination of structured die for 3D printing with fiber drawing allows to directly fabricate hundreds of meter of THz-guiding MPOF in a single step without any need for fiber preform fabrication, material preparation, and complex fiber drawing. Spectrogram analysis performed on different lengths of fibers prove singlemode operation and allow to determine the propagation loss.

### Suspended-core microstructured polymer optical fiber extrusion using 3D printers

Fused deposition modeling (FDM) 3D printers have been developed and optimized for the manufacturing of 3D model parts from a variety of materials including polymers, soft-glasses, and silica. The advanced control systems in the FDM 3D printers include built-in heating elements with temperature controllers and accurate material feeding systems provide the possibility to use the FDM 3D printers as fiber drawing towers^33^. Compared with other techniques used to fabricate optical fiber preforms, extrusion is a single-step technique that has significant potential for the fabrication of soft glass and polymer optical fiber preforms with non-geometric patterns^34–38^. To manufacture the optical fiber preforms using extrusion, a soft bulk polymer or soft glass billet is forced through a die to create a preform with a complex transverse profile, complementary to that of the die. Non-circular holes, large air-filling fractions, and long fiber preform lengths, can be achieved by using extrusion. However, this technique is limited to the fabrication of structured optical fiber preforms. To fabricate the microstructured optical fibers (MOFs), fiber drawing using a drawing tower is still required.

In this work, the FDM 3D printer was used to extrude and draw MPOFs with a simple geometry (SC-MPOF) to allow for ease of production from a single material^39^. The nozzle of the 3D printer was modified and designed to have a complementary structure to the fiber cross-section. The structured nozzle was mounted on a 3D printer head, where heat from the heater cartridge could be transferred to the nozzle. A cyclic olefin copolymer (TOPAS) was the material of choice because of its low absorption coefficient, about 100 times lower than that of commercially available filaments, such as acrylonitrile butadiene styrene (ABS)^16^. The TOPAS (8007S-04) filament was produced by the Institute for Material Science and Plastics Processing, University of Applied Sciences Eastern Switzerland, Rapperswil, 8640, Switzerland, where a filament was extruded and drawn to the desired diameter of 2.85 mm. TOPAS 8007S-04 has a glass transition temperature (T_g_) and a melting point (T_m_) of 70–185 °C and 190–250 °C, respectively. When the polymer is heated to a temperature higher than T_g_, the relative position of molecular chains allow for a greater flexibility and the polymer transitions from solid to rubber. The surface roughness and defect formation of the extruded polymeric optical fiber work piece are strongly dependent on the fictive temperature. Extrusion parameters, including feed rate and nozzle temperature, were varied to investigate their effect on the transparency and surface roughness of the extruded fiber and to find the fictive temperature for optimal fiber drawing. The optical quality of the extruded polymer was evaluated by extruding the filament through a nozzle without subsequent drawing. During the process, the nozzle temperature (T_n_) was varied from 230 to 260 °C, and at T_n_ ~ 240 °C the surface of the polymer became smooth and shiny, with significantly reduced bubble formation compared to other nozzle temperatures. Bubbles occur when the air trapped in the filament, due to high temperature, begins to expand, and are detrimental as they disrupt the thin and delicate MPOF structure and result in fiber break during drawing.

The feed rate of the filament influences the temperature experienced by the polymer: slow filament feed rates result in high filament temperatures, while rapid rates cause low filament temperatures. Heater temperature and polymer feed rate were determined empirically from the fiber surface quality. Initially, a feed rate of s ~ 50 mm/min was selected resulting in air bubbles and rough surface due to excessively high fictive temperature. The extrusion velocity was then gradually increased and an extruded fiber blank with a smooth surface was successfully achieved at s ~ 180 mm / min and T_n_ ~ 240 °C.

**Figure 1:**
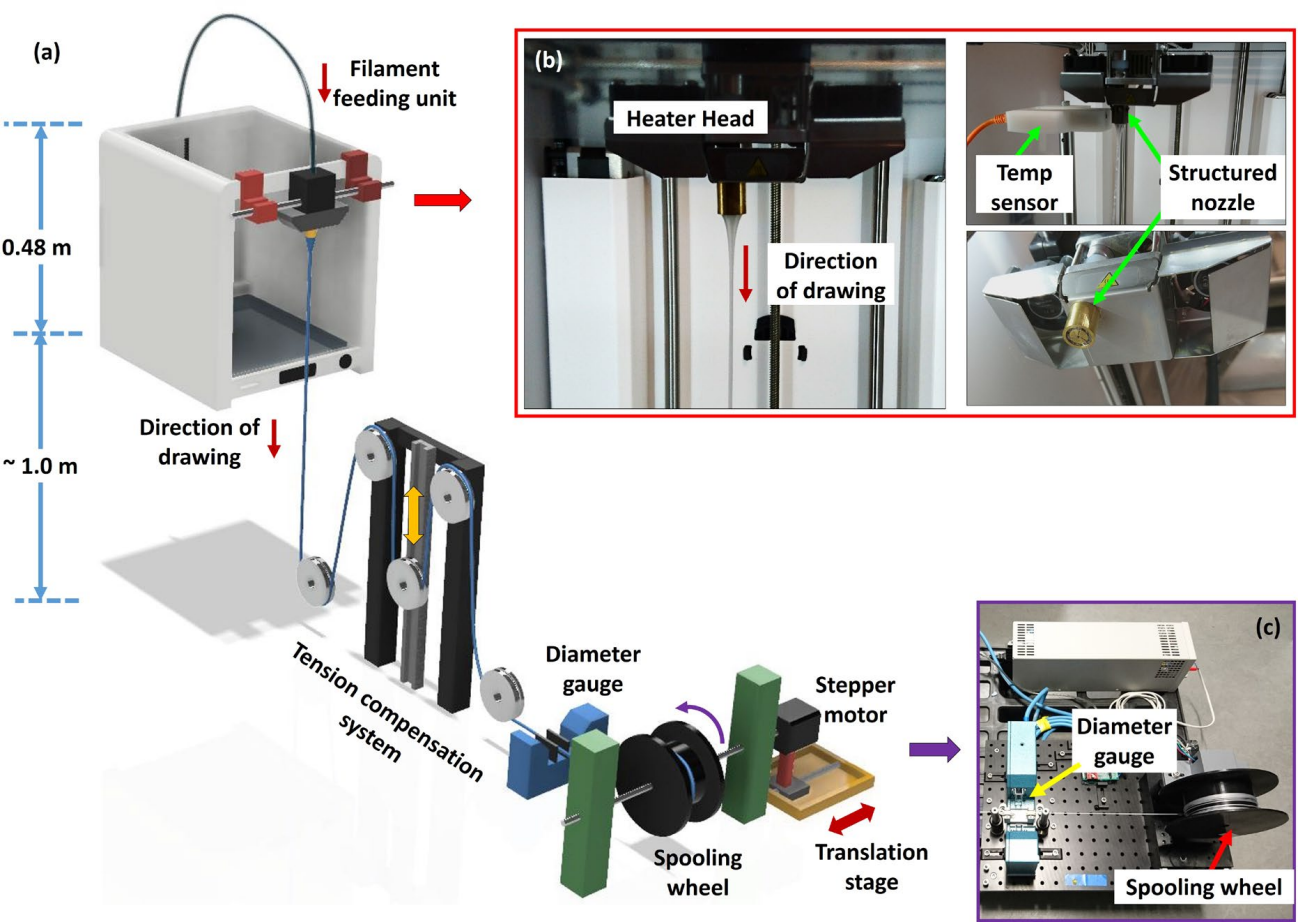
(**a**) Schematic of novel optical fiber drawing tower used to extrude the suspended-core microstructured polymer optical fiber. Experimental setup with (**b**) a close-up view of 3D printer heater head and a structured nozzle. (**c**) A fiber pulling system and the diameter gauge.

The TOPAS SC-MPOF was fabricated by feeding the filament using a built-in feeding motor operating at S ~ 180 mm/min, corresponding to an extrusion speed of 0.84 mm/s. To further reduce the SC-MPOF diameter, the extruded structured polymer fiber preform was connected to a spool (10 cm diameter) which was rotated at a constant speed by a stepper motor. The speed of fiber drawing was controlled by varying the rotation speed of stepper motor. The fiber diameter was monitored in real time during drawing by an optical diameter gauge. In this experiment, the spool was rotated with the rotation speed of 4 rpm, resulting in a fiber drawing speed of 21 mm/s. The size of the extruded fiber preform prior to drawing was 8 mm, while the final fiber diameter (d) was d ~ 1,600 µm. Figure 1a–c show the microstuctured fiber drawing tower which includes the FDM 3D printer head.

**Figure 2:**
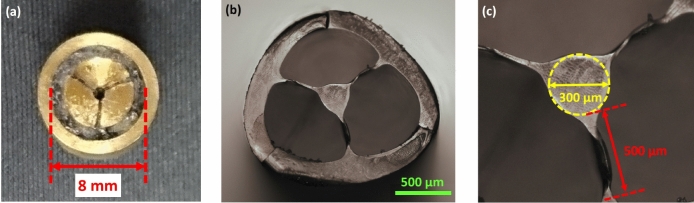
(**a**) Bottom view of the structured nozzle used to fabricate the SC-MPOF. (**b**) Cross-section of the TOPAS SC-MPOF with outer diameter d ~ 1,600 µm. (**c**) Close-up view of fiber core showing a 300 µm fiber core and 500 µm long strut.

To observe the cross-section, the SC-MPOF was cleaved using a heated razor blade. Figure 2a,b show the bottom view of the structured nozzle used to fabricate SC-MPOF and the cross-section of drawn SC-MPOF, respectively. By comparing the microscope images, it is possible to see that the microstructure inside the fiber was maintained after drawing. The core is optically isolated from the outer polymer region by three fine supporting struts. The pulling force between core and outer polymer region through the three supporting struts leads to the deformation of the circular core into a triangular core. The SC-MPOF outer cladding, core diameter, strut length and thickness were measured from cross-sectional microscope images imported into the ImageJ software. For the SC-MPOF triangular core profile, the measured core diameter is defined as the diameter of the largest circle that can be inscribed in the core region (Fig. 2c). The fiber with d ~1600μm had the core diameter of d_c_ ~ 300 μm, and strut 500 μm long and 25 μm thick. A large ratio of strut length to core diameter in the fiber is needed to prevent leakage of the guided mode into the cladding. The surface roughness observed in the extruded preform was further reduced by the fiber drawing and most of the bubbles inside the fiber preform disappeared. The improvement in the surface roughness of drawn polymer optical fiber compared with the extruded preform is a result of the realignment of the polymer chain during fiber drawing process. The contact between soft polymer and the nozzle inner surface is believed to be the source of the large surface roughness observed on the outer cladding of the extruded fiber preform, and polymer reflow during fiber drawing mitigates this effect, reducing corrugations on the surface and minimizing bubbles trapped in the polymer. The crack observed in the outer cladding of fiber cross-section (Fig. 2b) has been attributed to the fiber cleaving. These cracks are 1 mm in length and occur only at the fiber tip. The cracks do not affect the main SC-MPOF structure and the fiber microstructure is preserved along the fiber length.

## Results and discussion

**Figure 3:**
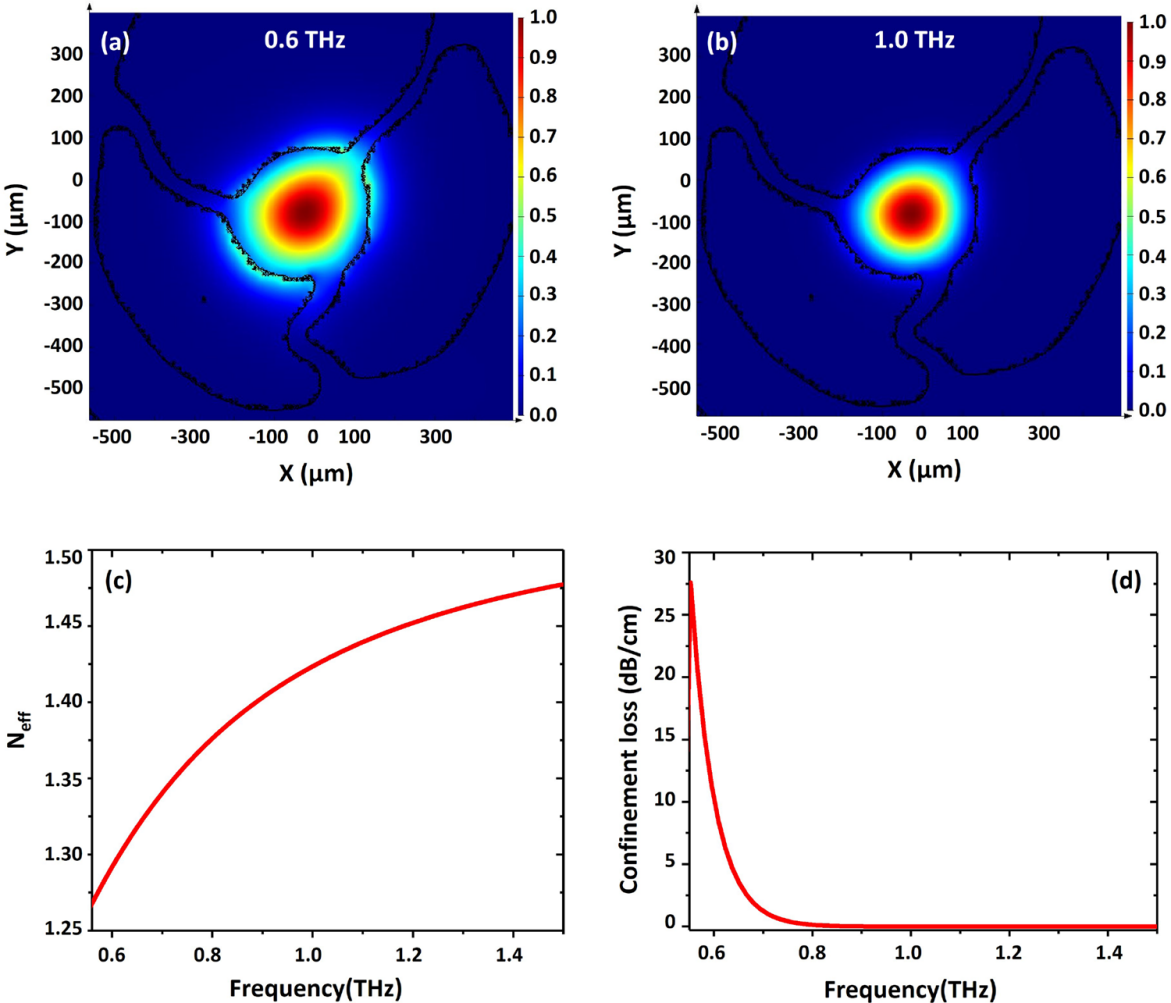
Simulated mode profiles in the fiber core at (**a**) ν = 0.6 THz and (**b**) ν = 1.0 THz. (**c**) Effective refractive index and (**d**) confinement loss of the fundamental mode at ν = 0.55–1.5 THz.

At these dimensions, the fiber is expected to operate in the single mode guidance in the frequency range ν ~ 0.5–1.0 THz. To confirm this, finite element simulations (using the commercial FEM solver Lumerical^©^ Mode Solution) were performed by importing the cross-section image of the fiber into the software. The mode analysis (Fig. 3a,b) reveals that the field is confined in the central solid core and guided by total internal reflection as illustrated by the output profiles at 0.6 THz and 1.0 THz. As expected, field confinement becomes stronger as the frequency increases such that for ν ~ 1.0THz the vast majority of the power propagates within the solid core. Figure 3c,d reveal the simulated effective refractive index (n_eff_) and confinement loss of the fundamental mode guided in the fiber core when the ν was scanned from 0.55 to 1.5 THz. Due to the larger mode field diameter at longer wavelengths, n_eff_ decreases for decreasing frequencies. The gradual decrease of n_eff_ at ν = 1.5–0.8 THz shows that the fundamental mode is mostly confined within the solid core. At ν ~ 0.8–0.55 THz, the mode expands more into the air holes surrounding the core resulting in a more pronounced decrease of n_eff_. The effect of mode field expansion at ν = 0.8–0.55 THz is even more evident in Fig. 3d, where the confinement loss of the fundamental mode increases dramatically. Simulations also show that the core does not support any other guiding mode at ν < 0.5 THz due to the large ration between wavelength (600 μm) and core diameter (300 μm).

**Figure 4:**
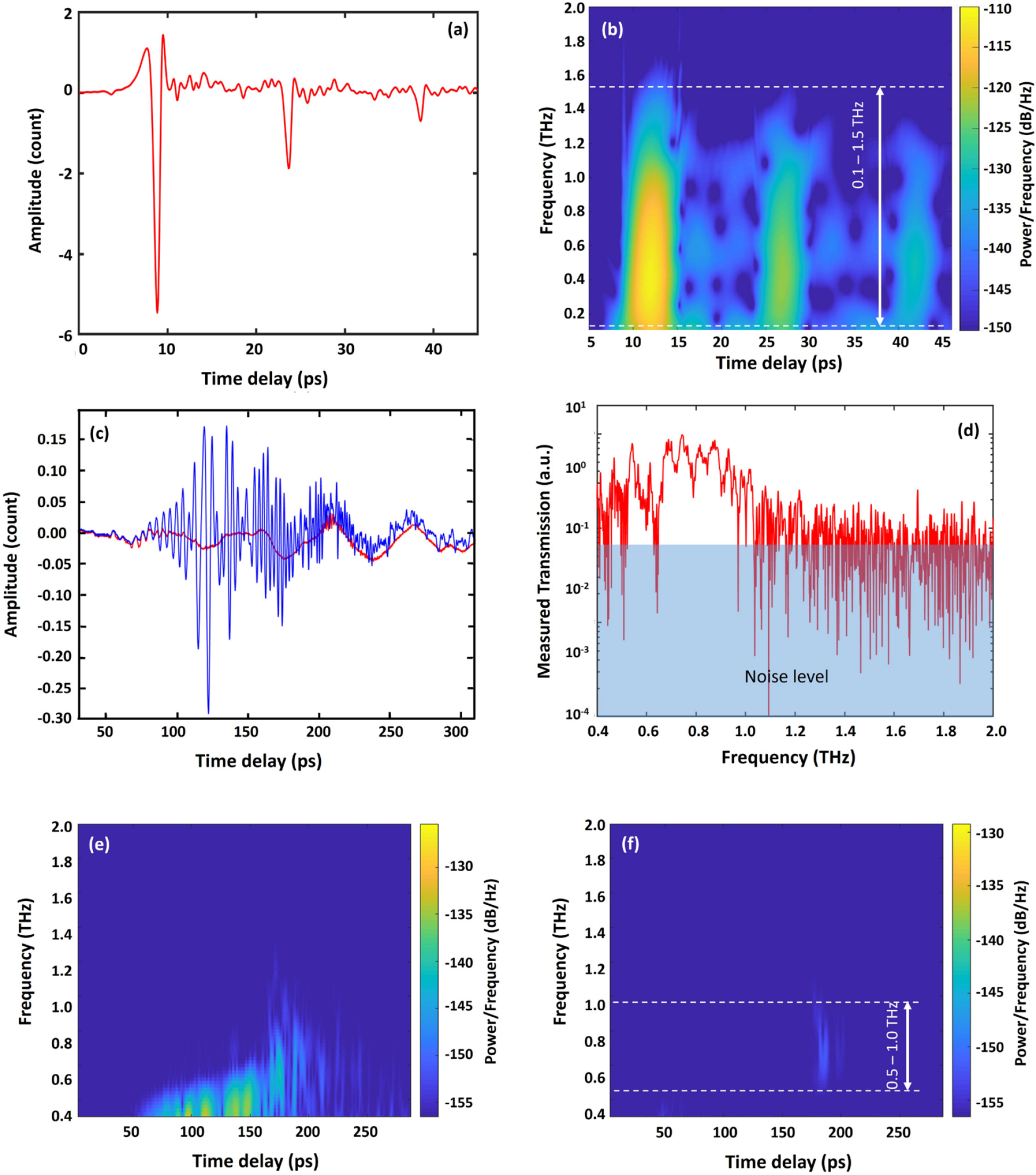
(**a**) Time domain measurement and (**b**) Spectrogram of the THz emitter. (**c**) Time domain measurement of the E-field of the THz waves transmitted through the TOPAS SC-MPOF; the blue and red lines represent the signal from the whole fiber (core and outer cladding) and from the fiber core only, respectively. (**d**) Spectral profile of the THz waves transmitted through the TOPAS SC-MPOF. Spectrogram of the THz wave guided (**e**) in the whole fiber cross section (outer cladding and core); and (**f**) fiber core only of an 80 mm long SC-MPOF.

Transmission in the THz part of the spectrum was characterized with a THz time domain (TD) setup. Pulses from a femtosecond laser at λ ~ 800 nm went through a beam splitter and while one part of the fs-laser beam was directed through an optical delay line used to adjust the propagating time of the wave, the other irradiated the THz emitter generating THz beam ^40^. A reference scan was performed without any optical fiber leaving the distance between the detector and the emitter unchanged. The time domain data and the time–frequency diagram (spectrogram) of the THz signal for these measurement are presented in Fig. 4a,b. This clearly shows that the effective bandwidth over which useful spectroscopic data was obtained lies in the range ν = 0.1–1.0 THz. The broadband THz wave was launched into an 8 cm long section of the SC-MPOF fiber using a THz focusing lens with focal length of 37 mm. The fs-laser wave from the time delay line and the transmitted THz wave from the fiber were simultaneously shone onto the THz detector to generate the signal using the THz-TDS detector. The time domain of transmitted wave from the fiber was recorded in real time and a subsequent Fourier transform in the time domain allowed to generate a transmission spectrum in the frequency domain.

In the focal point, the THz beam waist diameter was estimated to be ~ 1 mm at the frequency of 1 THz, comparable to the outer diameter of the SC-MPOF fiber. The microscope image in Fig. 3b shows that the cladding has an outer shell thickness of 200 μm, which supports THz wave propagation at ν = 0.5–1.0 THz. Cladding modes were removed by coating the fiber with graphite paste. High frequency signals in the time domain at time delays of *t ~ *60–180 ps were observed (Fig. 4c) prior to the removal of the cladding modes, and were ascribed to the multimode guidance in the outer cladding. These cladding modes were stripped by applying to the fiber outer surface graphite paste , which exhibits higher refractive index than most of polymers^41^, resulting in the disappearance of the high frequency signal, as shown in Fig. 4c (red line). The transmission spectrum of THz wave guided in the fiber core indicates that at ν ~ 1.0 THz, the output intensity is higher than the noise level (Fig. 4d), while it drops to the noise level at ν < 0.5 THz, confirming core guiding in the spectral range of ν ~ 0.5–1.0 THz, in accordance with simulations. The graph in Fig. 4c shows that the signal from the fiber is significantly decreased due to the cladding mode removal. The signal to noise ratio in singlemode operation is ~ 7 dB, which is acceptable to perform measurements in the THz regime.

The time–frequency diagram clearly demonstrates guidance in the TOPAS SC-MPOF (Fig. 4f). The spectrogram is calculated from the measured time trace of the THz output pulses using a sliding Gaussian-shaped sample window of 128 samples. The windowed time domain scans are then Fourier transformed into the frequency domain, with spectral components plotted against the window position in time. The spectrograms show few qualitative features of the fibers: Fig. 4e demonstrates that before removing the cladding modes, there is evidence of dispersion at ν ~ 0.4–1.2 THz, as seen by the earlier arrival of the low frequency components. This dispersion is associated to the modal dispersion of multimode guiding. After cladding modes were stripped, the spectral components of the pulse aggregate at around the arrival time *t* ~ 180 ps (Fig. 4f). Higher-order modes have effective phase indices, lower than that of the fundamental mode, which would result in several temporally displaced islands of spectral components. Absence of this effect indicates that the wave in the fiber core is guided in a single mode regime. The pulse has negligible dispersion indicated by the simultaneous arrival of all frequency components. The zero-dispersion frequency range is estimated to be at ν ~ 0.5–1.0 THz, centered at ν ~ 0.8 THz. The low frequency components (ν ~ 0.4–0.6 THz), arriving at *t* ~ 50–150 ps, disappear when the modes guided by outer cladding have been removed, confirming that these are high order modes guided in the outer cladding. The temporal components arriving after 200 ps also disappear in the graphite coated fiber and are also ascribed to the index guided modes in the outer cladding.

**Figure 5:**
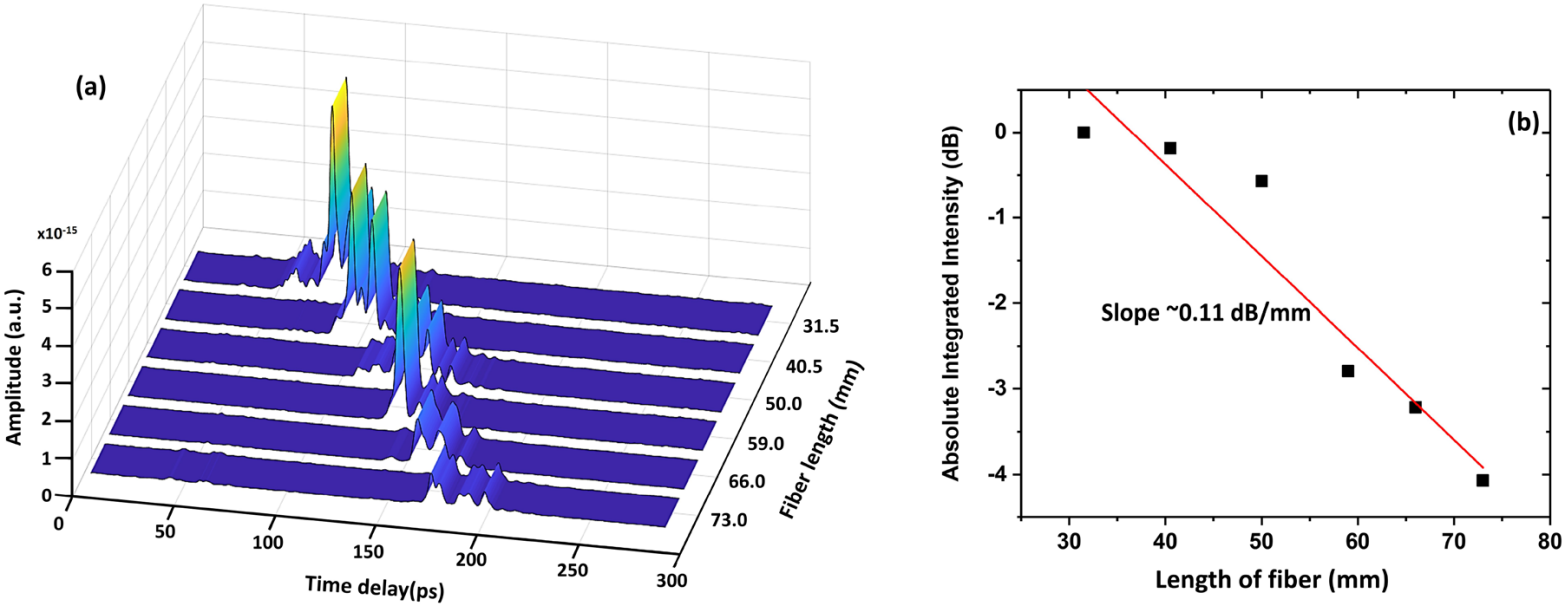
(**a**) Temporal profile of THz wave after propagation through different lengths of SC-MPOF. (**b**) Relationship between THz transmission and fiber length.

Fiber propagation losses were estimated using the cut-back method. The length of the fiber was gradually shortened from 73 to 31 mm by using a razor blade. The transmission of the THz wave propagated through the fiber of different lengths was measured using a Meno GMBH TERA8-1 photoelectric antenna. The resulting data was analyzed using a spectrogram, which provides a clear view of the delay occurring due to different fiber lengths. Signal intensity was calculated by integrating all spectral components in the ν ~ 0.5–1.1 THz range (Fig. 4f) for each fiber length. The result shows that the THz pulse arrives at the detector at t ~ 180 ps for the longest fiber (73.0 mm), and at t ~ 80 ps when propagating through the shortest fiber (31.5 mm). The graph clearly shows a drop in the signal amplitude as the fiber length increases. The effective index (n_eff_) of fundamental mode guided in fiber core can be calculated from the result in Fig. 5a. The result show that the calculated n_eff_ of this mode is ~ 1.428, in agreement with the value obtained in the simulations of Fig. 3c (n_eff_ ~ 1.424). The average propagation loss was 0.11 dB/ mm across the frequency range ν = 0.4–1.1 THz (Fig. 5b).

## Conclusions

In conclusion, the direct drawing of microstructured polymer optical fibers using a cheap desktop 3D printer has been demonstrated. Given the relatively low cost and ease of operation, compared to a conventional drawing tower, current desktop 3D printers may become an invaluable tool for the production of microstructured optical fibers. Analysis of the fiber produced during this work proves the ability to maintain the microstructure inside the fiber after the drawing from a customized 3D printer head. The near field and modal profile simulations demonstrate the ability to confine light in the fiber core in the spectral range ν = 0.5–1.0 THz. Time and frequency domain spectrograms confirmed core guidance and allowed to measure an average propagation loss of 0.11 dB/mm at ν = 0.4–1.1 THz. The delivery of the THz beam to enclosed locations such as chemical reactors are difficult in free space, so the ability to confine the beam to a bendable fiber is extremely beneficial even if at the expense of relatively large losses. By improving the launching conditions and the fiber structure symmetry, the loss can be further reduced, and this will be pursued in future works.

## Material and methods

**Figure 6:**
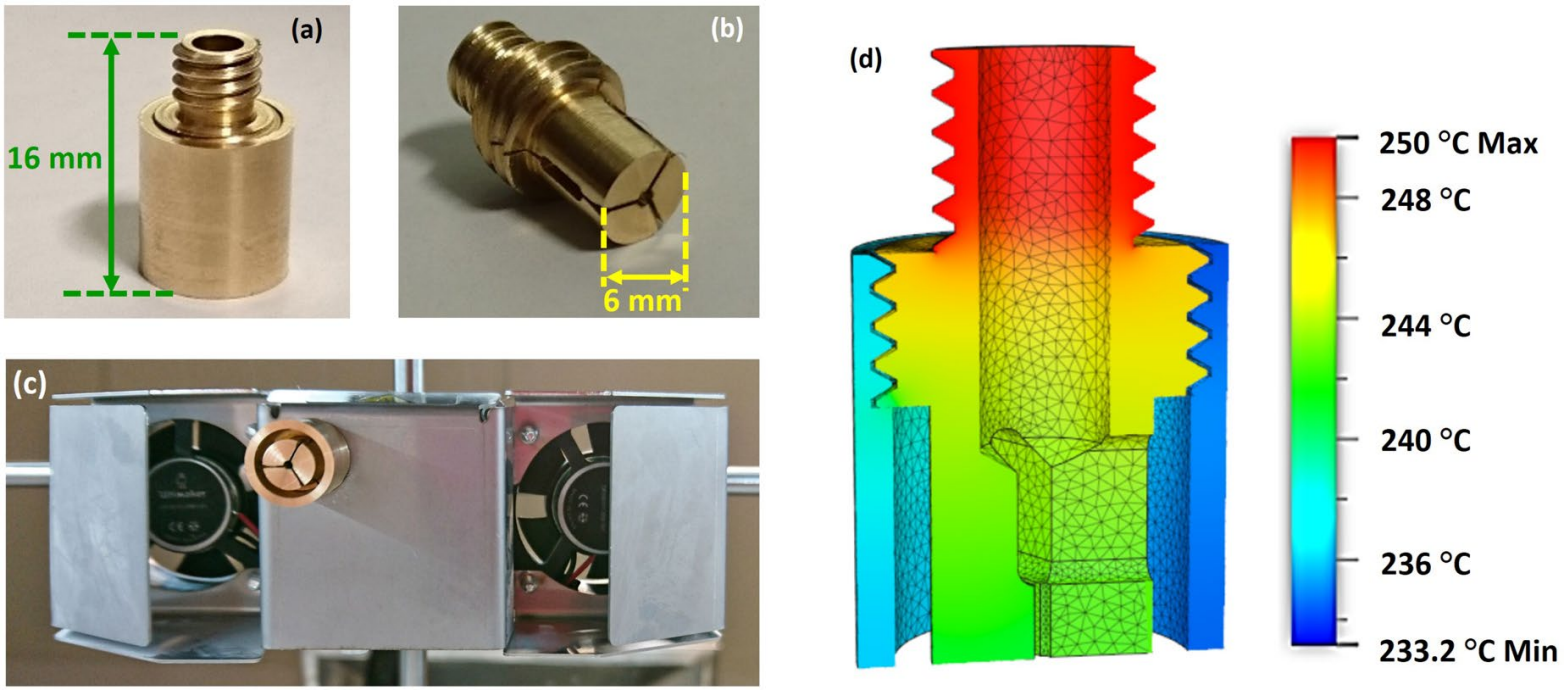
Micromachined structured nozzle: (**a**) body and cover side-view, (**b**) body side-view, (**c**) structured nozzle connected with the 3D printer at the heater head. (**d**) Heat transfer simulation of the designed nozzle when the temperature of 250 °C has been applied to the nozzle thread.

An extrusion die (nozzle) with the reciprocal structure of the SC-MPOF cross section was designed by using the Autodesk software (Fusion 360). The structured nozzle design was separated into two pieces: the structured body and the cover. The standard size of the polymer filament for the 3D printer used in this experiment is 2.85 mm in diameter. The body of nozzle has a 3.0 mm aperture diameter at the input, where the polymer is fed into the nozzle. The structured body of the nozzle shapes the extruded fiber, resulting in a solid suspended core connected by three struts to the outer cladding. The cover is a tube with an inner diameter of 8.0 mm and outer diameter of 10.0 mm. The gap between the cover and the structured body determines the outer cladding thickness in the extruded SC-MPOF. Figure 6a,b shows various views of the structured nozzles. The standard 3D printer nozzle has been replaced with the structured nozzle by connecting the structured nozzle with the heater block at the 3D printer heater head (Fig. 6c). Heat from the heater cartridge (24 V, 35 W) was applied to the upper thread of the nozzle via the heater block. Heat transfer simulations of the structured nozzle were used to optimize the nozzle length and showed (Fig. 6d) that the temperature at the nozzle bottom is reduced from the applied temperature of 250.0–233.2 °C when the nozzle length is 16 mm. This temperature is in the range required for printing the TOPAS filament (230–250 °C). The 3D model design was used for computer numerical control (CNC) the nozzle. Indeed, micromachining is a technique traditionally used to fabricate the dies for the extrusion of structured fiber preforms.

**Figure 7:**
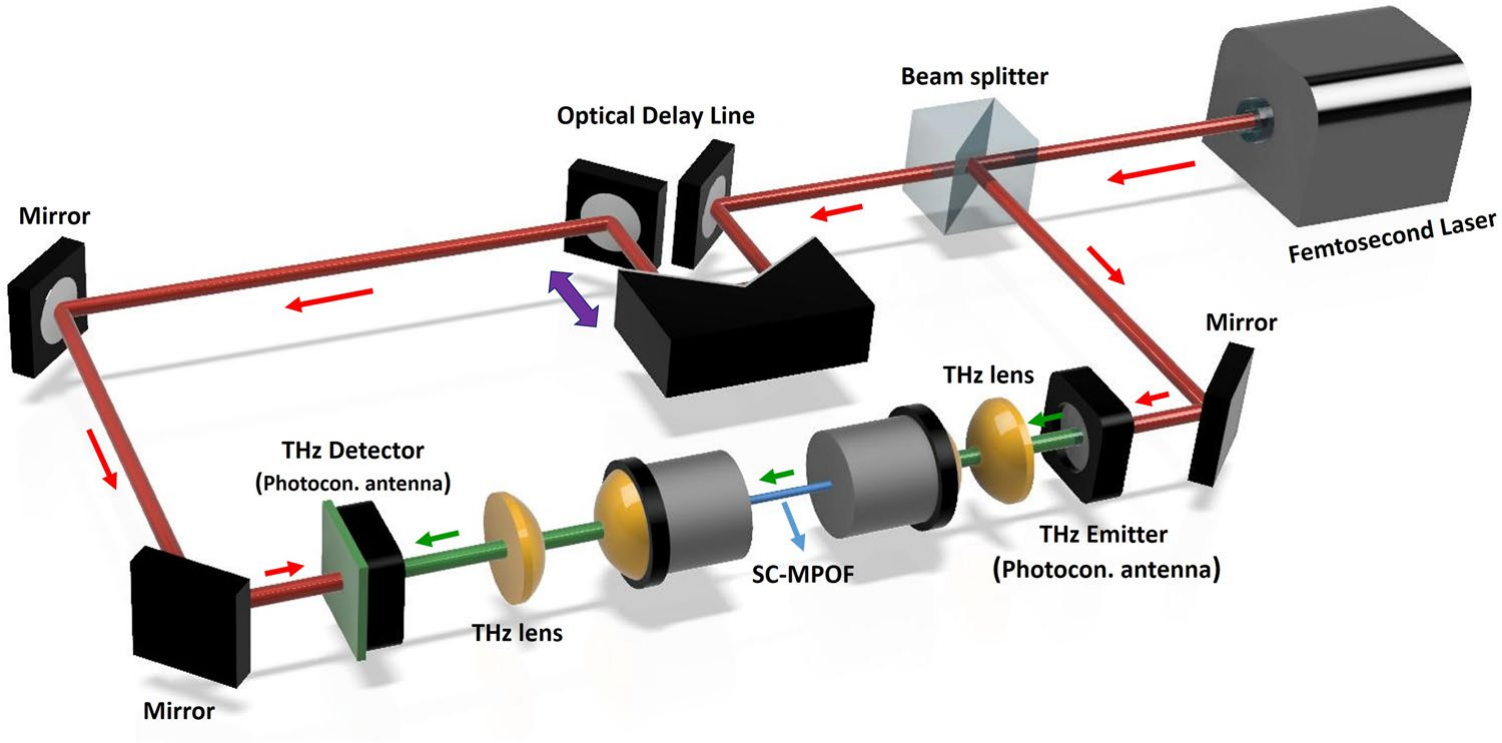
Schematic of the experimental setup used for THz characterization of the extruded TOPAS SC-MPOF.

The optical properties of TOPAS SC-MPOF were characterized using THz time-domain spectroscopy (THz-TDS) (Fig. 7). To ensure a constant amount of light coupled to the fiber, the SC-MPOF was mounted in a custom-made holder with lenses attached at the ends. The holder allowed the input end of the fiber to be securely fixed at the focal point of the focusing lens. Another lens with a focal length of 37 mm was inserted at the other end of the holder and designed for collimation of the outgoing fiber. The THz source used in the experiment (Laser Quantum GMBH's Tera-SED with GaAs photoconductor) emitted broadband THz waves up to ν ~ 2.5 THz. The detector function was performed by the Menlo GMBH TERA8-1 fiber optic antenna with a bandwidth of up to 4 THz. Although the source and detector are active up to ν ~ 2.5 THz and ν ~ 4 THz, respectively, the spectroscopic bandwidth is limited by ambient noise and component misalignment, which in particular limits the high end of the frequencies.
